# A new prognostic model based on gamma-delta T cells for predicting the risk and aiding in the treatment of clear cell renal cell carcinoma

**DOI:** 10.1007/s12672-024-01057-2

**Published:** 2024-05-25

**Authors:** Yaqian Wu, Mengfei Yao, Zonglong Wu, Lulin Ma, Cheng Liu

**Affiliations:** 1 Department of Urology, Peking University Third Hospital, Beijing, 100191 People’s Republic of China; 2 Department of Pathology, National Cancer Center/National Clinical Research Center for Cancer/Cancer Hospital, Chinese Academy of Medical Sciences and Peking Union Medical College, Beijing, People’s Republic of China; 3 Department of Urology, Shanghai General Hospital, Shanghai Jiao Tong University School of Medicine, Shanghai, 200080 People’s Republic of China

**Keywords:** Clear cell renal cell carcinoma, γδ T cell-related genes, Risk score, Prognosis, TMSB10

## Abstract

**Background:**

ccRCC is the prevailing form of RCC, accounting for the majority of cases. The formation of cancer and the body's ability to fight against tumors are strongly connected to Gamma delta (γδ) T cells.

**Methods:**

We examined and analyzed the gene expression patterns of 535 individuals diagnosed with ccRCC and 72 individuals serving as controls, all sourced from the TCGA-KIRC dataset, which were subsequently validated through molecular biology experiments.

**Results:**

In ccRCC, we discovered 304 module genes (DEGRGs) that were ex-pressed differentially and linked to γδ T cells. A risk model for ccRCC was constructed using 13 differentially DEGRGs identified through univariate Cox and LASSO regression analyses, which were found to be associated with prognosis. The risk model exhibited outstanding performance in both the training and validation datasets. The comparison of immune checkpoint inhibitors and the tumor immune microenvironment between the high- and low-risk groups indicates that immunotherapy could lead to positive results for low-risk patients. Moreover, the inhibition of ccRCC cell proliferation, migration, and invasion was observed in cell culture upon knocking down TMSB10, a gene associated with different types of cancers.

**Conclusions:**

In summary, we have created a precise predictive biomarker using a risk model centered on γδ T cells, which can anticipate clinical results and provide direction for the advancement of innovative targeted therapies.

**Supplementary Information:**

The online version contains supplementary material available at 10.1007/s12672-024-01057-2.

## Introduction

Renal cell carcinoma (RCC) is a highly prevalent cancer, consistently ranking among the top ten most common cancers worldwide. It specifically comes from cells of the renal tubular epithelium [1]. ccRCC, the most prevalent and lethal form of RCC, is responsible for the majority of fatalities associated with kidney cancer [2]. Localized RCC is best treated with nephrectomy, which is linked to a positive clinical outlook. Nevertheless, the outlook for advanced or metastatic renal cell carcinoma (mRCC) is typically bleak [3], and there has been a gradual rise in the incidence of metastatic clear cell renal cell carcinoma (ccRCC) in recent decades. Moreover, the survival rate of ccRCC patients is less than ideal due to genetic resistance to traditional chemotherapy and radiotherapy, as well as acquired resistance to targeted treatments [4]. Continued investigation of ccRCC pathogenesis is crucial in order to discover new markers and potential therapeutic targets for ccRCC.

The tumor growth and progression in ccRCC are influenced by various characteristics that indicate the involvement of immune-mediated mechanisms. Administering recombinant IL-2 or IFN-α can impede the advancement of solid tumors in individuals with ccRCC [5]. Moreover, the direct proof of an immune response development in ccRCC is demonstrated by the therapeutic effectiveness of systemic cytokine therapy [6]. Treating ccRCC can potentially be accomplished through the use of T cell therapy.

While not undergoing clonal proliferation, these T cells play vital roles in immune monitoring, immune regulation, and effector activities [7]. Recent research indicates that γδ T cells play a crucial role in anti-tumor immunosurveillance due to their strong an-ti-tumor capabilities and distinct functions [8, 9]. Research conducted in both clinical and preclinical settings suggests that T cells possess the potential to serve as a promising therapeutic modality for various malignancies, including ccRCC [10–13]. Only a few studies have examined the predictive significance of T lymphocytes in ccRCC. Hence, it is imperative to investigate a novel gene associated with γδ T cells to effectively monitor the development and advancement of tumors.

In the present study, a total of 13 DEGRGs were identified as having prognostic significance in ccRCC. Additionally, a risk model was constructed to assess the likelihood of survival in ccRCC patients, incorporating the risk score, age, and N stage. Furthermore, a nomogram was devised to predict the survival outcome in ccRCC patients based on these aforementioned factors. The potential of risk signatures to predict immune infiltration and therapy effectiveness has also been examined. In summary, our investigation has revealed the significant involvement of β-Thymosin 10 (TMSB10) in both immune response and tumor progression. Although TMSB10 has been identified as an oncogene in various cancer types, its specific role in clear cell renal cell carcinoma (ccRCC) remains uncertain and necessitates additional exploration [14–16]. The outcomes of our study indicate a potential association between TMSB10 and the infiltration of immune cells within tumors, subsequently influencing the prognosis of ccRCC patients. Moreover, it might be a viable target for cancer therapy.

## Materials and methods

### Data source

The training cohort consisted of clinical data and RNA sequencing data obtained from The Cancer Genome Atlas Program (TCGA-KIRC, https//portal.gdc.cancer.gov) database. The study incorporated the information from every one of the 535 ccRCC tumor samples as well as 72 samples of healthy kidneys. The TCGA-KIRC dataset contained clinical data on ccRCC patients. Furthermore, the validation cohort consisted of 101 ccRCC samples obtained from the Array Express database, specifically from the E-MTAB-1980 cohort.

### Acquirement of differentially expressed genes

R package “DEseq2” was used to was utilized to detect genes that were expressed differently (DEGs) between ccRCC and control samples with |log2FC|> 1 and adj.P.Val < 0.05 [17].

### Analysis of immune infiltration

CIBERSORT, a deconvolution tool, employs linear support vector regression to as-certain the expression matrix of different subtypes of human leukocytes [18]. Using the R package called 'CIBERSORT', we calculated the ratios of the 22 immune cells that infiltrated each sample. Next, we computed the correlation among immune cells. Prominent findings (p < 0.05) were chosen for further examination.

### Weighted gene co-expression network analysis (WGCNA)

Using the WGCNA package of R software [19], we created a weighted co-expression network for the expression profile data of the TCGA-KIRC dataset DGEs and γδ T cell-related module genes were overlapped to obtain the differentially expressed γδ T cell-related genes (DEGRGs). Afterwards, we utilized the 'maftoools' R package to visualize the somatic mutation data in Mutation Annotation Format (MAF) and discovered the top 20 genes with a high mutation rate among DEGRGs.

### Functional annotation and pathway enrichment analysis

DEGRGs underwent functional enrichment analysis in three domains of Gene Ontology (GO) [20], namely biological process (BP), cellular component (CC), and molecular function (MF). The KEGG database [21] contains datasets of pathways involving biological functions, diseases, chemicals, and drugs. An enrichment analysis was con-ducted using the “clusterProfiler” R package to determine the biological functions of the genes and associated pathways. Adj.p < 0.05 was considered statistically significant.

### Establishment and validation of the prognostic model

In TCGA-KIRC, the connections between the DEGRGs and the overall survival (OS) of patients were assessed using univariate cox regressions. Further analysis was con-ducted on all genes that had HR ≠ 1&p < 0.01 after screening. Afterwards, the LASSO analysis was conducted to further choose prognostic genes associated with OS, and risk scores were built. The risk score was computed using the given equation: risk score = ∑ (Coef j * Xj), where Coef j represents the coefficient obtained through multivariate cox and Xj represents the messenger RNA (mRNA) expression of DECRGs. Based on the risk score median, the patients were divided into groups of high-risk and low-risk individuals.

Afterwards, we conducted univariate and multivariate cox regression analyses to determine if the risk scores and relevant clinical parameters could serve as independent predictors for ccRCC patients. The calibration curve was used to assess the performance of the nomogram.

### Evaluation of the immune microenvironment landscape

Predicting the immune and stromal components in tumors or diseases can be done using the immune scores and stromal scores. The ESTIMATE algorithm [22], available in the R package 'ESTIMATE', was used to calculate the immune and stromal scores of ccRCC samples.

### Evaluation of the therapeutic efficacy

We employed computational techniques to analyze the impact of immunotherapy in order to assess the therapeutic sensitivities of the two risk groups. The immunophenoscore (IPS), obtained from The Cancer Immunome Atlas (TCIA, https://tcia.at), has shown its ability to forecast patients' reaction to immune checkpoint inhibitor (ICI) therapy. A superior IPS score signifies an improved immunotherapy reaction. In addition, Tumor Immune Dysfunction and Exclusion (TIDE, http://tide.dfci.harvard.edu/), has been developed to assess mechanisms of immune evasion and to predict immunotherapy response.

### Cell culture

The American Type Culture Collection (ATCC) provided the human clear cell renal cell carcinoma cell lines (786-O and 769-P) for purchase. The cell lines were grown in RMPI-1640 medium (Gibco, Carlsbad, USA) supplemented with 10% FBS (Gibco, Carlsbad, USA). The cell lines were cultured in environment with 5% CO_2_ at a temperature of 37 °C.

### siRNA interference assay

Umine bioTechnology (Guangdong, China) provided the purchased TMSB10-specific siRNAs. The control was provided by using scrambled siRNA. The sequence of the siRNA was as stated below:

si- TMSB10-1 sense: 5′-CAGGAGAAGCGGAGUGAAAUUTT-3′,

antisense: 5′-AAUUUCACUCCGCUUCUCCUGTT-3′;

si- TMSB10-2 sense: 5′-CCCAGUCGUGAUGUGGAGGAATT-3′,

antisense: 5′-UUCCUCCACAUCACGACUGGGTT-3′;

si- TMSB10-3 sense: 5′-CGACCAAAGAGACCAUUGATT-3′,

antisense: 5′-UCAAUGGUCUCUUUGGUCGTT-3′;

si–N.C sense: 5′-UUCUCCGAACGUGUCACGUTT-3′,

antisense: 5′-ACGUGACACGUUCGGAGAATT-3′.

Lipofectamine RNAiMAX Reagent from Invitrogen was used to transfect ccRCC cells with siRNA.

### Quantitative real-time RT-PCR

The RNA Isolation kit (R0024, Beyotime, China) was utilized to extract the entire RNA, which was then quantified following the previously described method. The relative RNA expression levels were calculated using the 2^− ΔΔCT^ method with GAPDH as an endogenous control. The PCR primers were TMSB10, 5-CGATAAGGCCAAGCTGAAGAAAACG-3 (forward) and 5-CACTCCGCTTCTCCTGCTCAATG-3 (reverse); GAPDH, 5-CAGGAGGCATTGCTGATGAT-3 (forward) and 5-GAAGGCTGGGGCTCATTT-3 (reverse), in the following sequences.

### Western blotting

Total protein from ccRCC cells was extracted using RIPA buffer (Beyotime Biotechnology, China). Then, Polyvinylidene difluoride (PVDF) membrane (Millipore, Billerica, MA, United States) was used for protein transfer. The primary antibodies of TMSB10 (1:200, santa cruz, sc-514309) and GAPDH (1:2000, Proteintech, 10494-1-AP).

### Cell proliferation assay

To perform the CCK-8 test, ccRCC cells were seeded at a density of 2 × 10^3^ cells per well in 96-well plates and cultured in RMPI-1640 medium containing 10% FBS. Based on the manufacturer's instructions, a Cell Counting Kit-8 (Dojindo, Japan) was used to measure the rate of cell growth. ccRCC cells were placed in 24-well plates for the EdU assay. Upon reaching 80% confluence, the BeyoClickTM EdU-488 kit (Beyotime, Shanghai, China) was employed to determine cell growth rate. Following the application of a stain, the cells were observed using a fluorescence microscope manufactured by Olympus in Tokyo, Japan.

### Cell migration and invasion assays

Cell migration and invasion were assessed using a Transwell assay with 8-μm inserts. In the Transwell upper chambers, 1 × 10^5^ cells were suspended in 200 μl of serum-free RMPI-1640 media and introduced, whereas the lower wells were filled with medium containing 20% FBS. The process of cell invasion resembled cell migration and the inserts were coated with Matrigel 1 mg/ml instead (BD Bioscience) and left to incubate at 37 °C for 30 min prior to introducing the suspended cells into the upper chambers. The migration and invasion chambers were left to incubate for a duration of 17 h and 30 h, respectively. The trials were conducted autonomously on a minimum of three occasions.

### Statistical analysis

To compare two groups, the Wilcoxon test was employed, while for comparing more than two groups, the Kruskal–Wallis test was utilized. The Kaplan–Meier log rank test was utilized to analyze the survival curves. Further analyses included the CIBERSORT algorithm results that had a p-value less than 0.05. Statistical significance was determined by a p-value less than 0.05, with a two-tailed test. Levels of significance were reported as *p < 0.05, **p < 0.01, ***p < 0.001, and ****p < 0.0001 respectively.

## Results

### Identification of DEGs

**Fig. 1:**
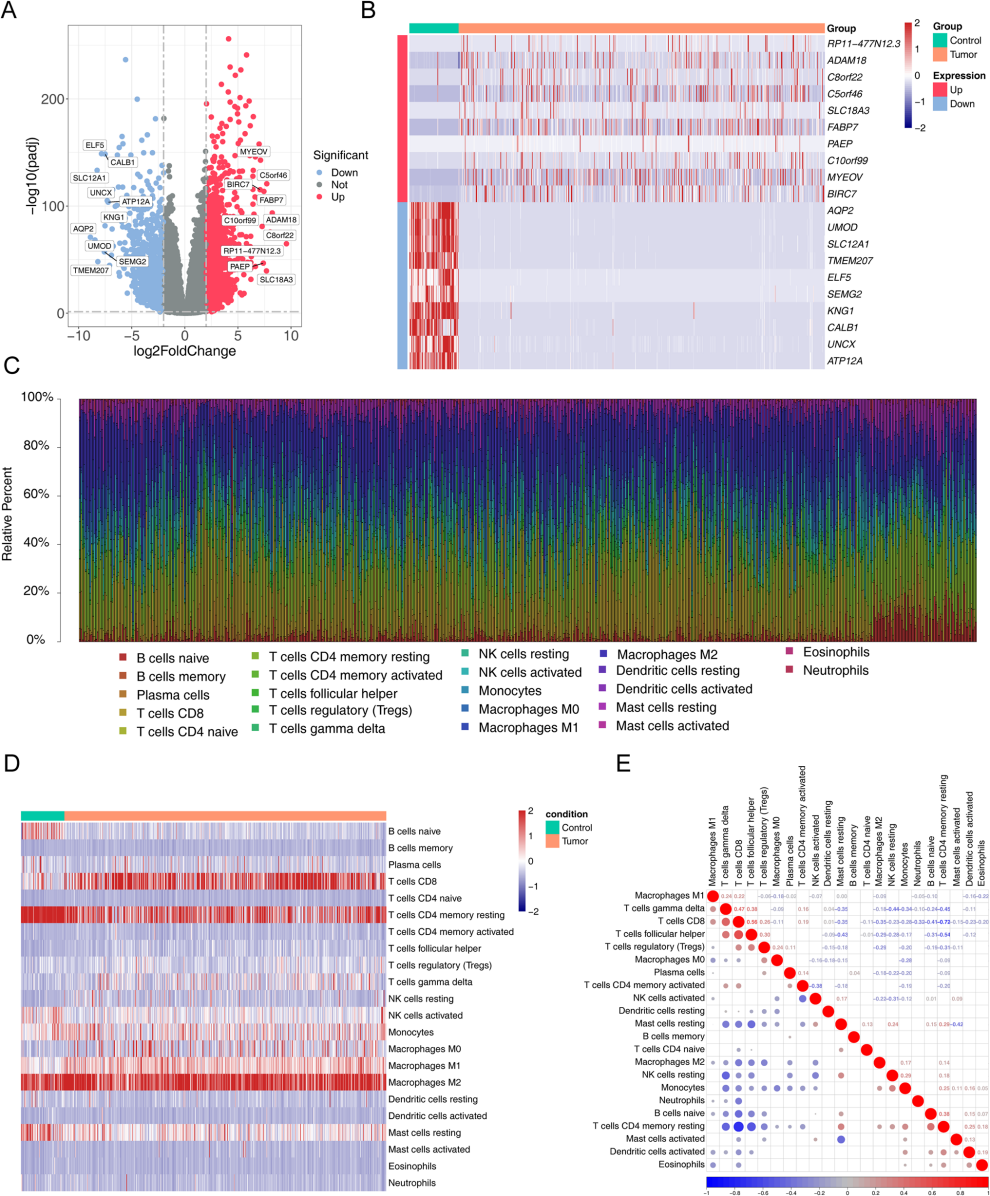
DEGs identification and Immune infiltration analyses. **A**, **B** The volcano plot and heatmap are used to show the top 10 up- and 10 down-regulated genes among the 2314 DEGs. **C** The distribution of 22 distinct subpopulations of immune cells is observed in each ccRCC sample. **D** A comparison is made between the immune cell infiltration scores of ccRCC patients and a control group. **E** Intrinsic correlation of 22 infiltrating immune cells in ccRCC

A comprehensive analysis identified a total of 2314 genes exhibiting differential expression, with 1508 genes being upregulated and 806 genes being downregulated. The selection of these differentially expressed genes (DEGs) was based on criteria of |log2FC|> 1 and adj. p < 0.05, as illustrated in (Fig. 1A). Furthermore, Fig. 1B presented a heatmap showcasing the top ten significantly upregulated and downregulated genes.

### Immune infiltration analyses

Figure 1C were employed to ascertain the proportional representation of 22 distinct subgroups of immune-infiltrating cells in each sample. A heatmap was created to summarize the ratings of immune cell penetration in individuals diagnosed with ccRCC and the comparison group (Fig. 1D). To obtain a deeper understanding of the possible inherent connections between invading immune cells, we conducted a correlation analysis to visually represent their extensive interactions (Fig. 1E). According to our analysis, a significant negative correlation (cor =  − 0.72) exists between CD4+ memory resting T cells and CD8+ T cells.

### Weighted co-expression network construction and identification of key modules

**Fig. 2:**
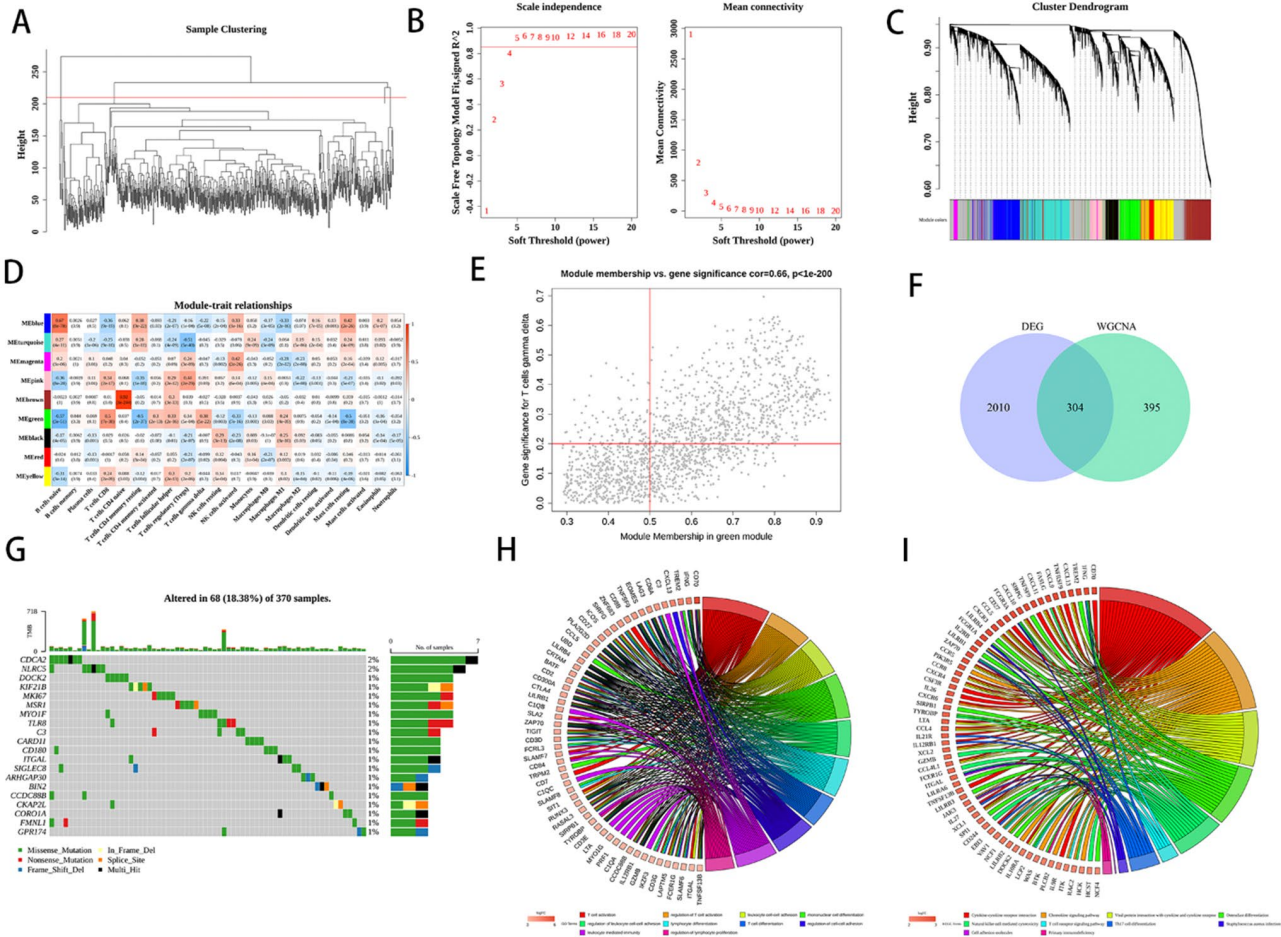
Construction of weighted co-expression network and analysis of DEGRGs. **A** Cluster tree for the 584 samples. **B** The ideal soft threshold for evaluating a network of scale-free co-expression. **C** A dynamic tree-cutting algorithm was used to combine related genes with comparable expression patterns into one module, resulting in a hierarchical clustering tree. **D** Heatmap of the correlations between the modules and immune-infiltrating cells (traits). **E** Module Membership in green module. **F** Venn diagram of DEGRGs. **G** The top 20 mutated genes of DEGRGs in ccRCC. GO** H** and KEGG **I **enrichment analysis of DEGRGs

The samples were clustered using the Pearson’s correlation coefficient. After removing any outliers, a cluster tree was generated for the remaining 584 samples (Fig. 2A). Figure 2B shows the application of a soft threshold of five in order to build a scale-free network. Using average hierarchical clustering and dynamic tree clipping, we were able to identify a total of nine modules (Fig. 2C). In the Fig. 2D, there was a significant association between the green module and γδ T cells. To ascertain relevant genes, a screening process was undertaken to identify genes with a gene significance (GS) value exceeding 0.2 and module membership (MM) value surpassing 0.5 (Fig. 2E). By overlapping the 699 module genes with the DEGs, we were able to acquire 304 DEGRGs (Fig. 2F). Furthermore, we identified the top 20 mutated DEGRGs in ccRCC with ranked percentages, and found increased somatic mutations in CDCA2, NLRC5, DOCK2, KIF21B, and MKI67 (Fig. 2G).

### GO and KEGG enrichment analyses of DEGRGs

GO functional analysis of the 304 DEGRGs identified involvement in T cell activation, regulation of T cell activation, and leukocyte cell–cell adhesion (Fig. 2H). KEGG pathway analysis of the 304 DEGRGs indicated significant associations with cytokine–cytokine receptor interactions, chemokine signaling pathways, and viral protein interactions with cytokines and cytokine receptors (Fig. 2I).

### Establishment and validation of the prognostic model

**Fig. 3:**
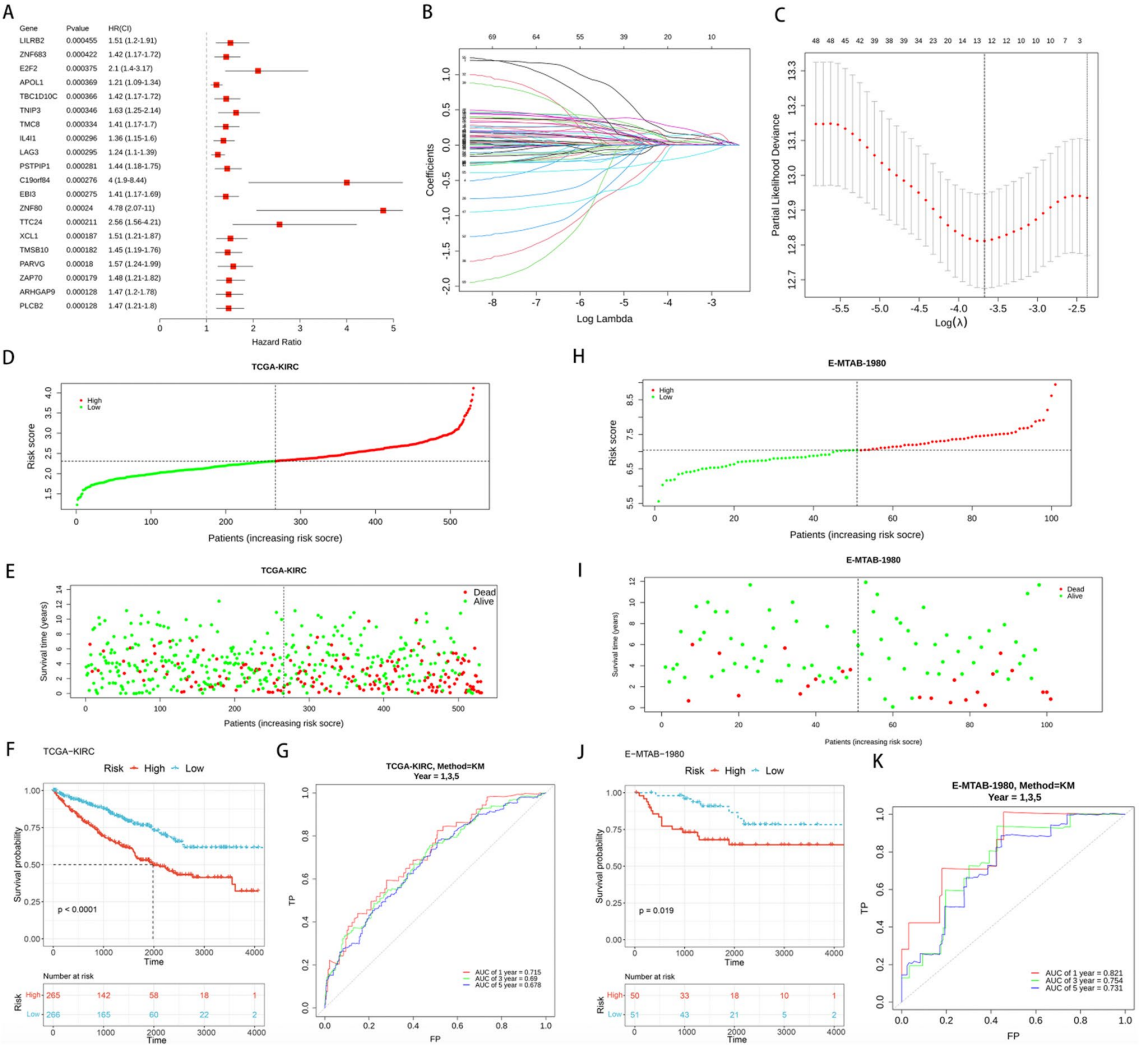
Establish and verify the prognostic model. **A** Univariate Cox regression analysis. **B**, **C** LASSO analysis with a minimum lambda value. In the training set: **D**, **E** Risk score and the survival status **F** Kaplan–Meier curves of the high- and low- risk groups (p < 0.0001). **G** ROC curve for 1-, 3-, 5-year survival predictions in patients with ccRCC based on the risk score. In the validation set: **H**, **I** Risk score and the survival status. **J** Kaplan–Meier curves of the high- and low-risk groups (p < 0.019). **K** ROC curve for 1-, 3-, 5-year survival predictions in patients with ccRCC based on the risk score

Analysis of the 304 DECRGs using univariate Cox regression revealed that 70 DEGRGs displayed a significant association with the OS of ccRCC patients (p < 0.2) (Fig. 3A). In order to reduce the effects of overfitting, we employed LASSO analysis to choose the most significant genes for our predictive model. We discovered and kept 13 genes that showed substantial predictive worth (Fig. 3B, C). Afterwards, we created a risk score equation for ccRCC patients by utilizing the expression levels of the previously mentioned 13 genes. The risk score can be calculated using the following formula: risk score = (0.159 × PLCB2 expression value) + (0.141 × TMSB10 expression value) + (0.055 × ZNF80 expression value) + (0.099 × IL4I1 expression value) + (0.058 × TNIP3 expression value) + (0.011 × C2 expression value) + (0.043 × PMCH expression value) + (0.241 × NUSAP1 expression value) + (0.032 × HSD3B7 expression value) + (0.030 × BATF3 expression value) + (− 0.178) × ENPP3 expression value) + (0.033 × NNMT expression value) + (0.005 × LHFPL2 expression value).

The risk score for each patient in the training and validation sets was computed by utilizing the established formula. Following this, we categorized them into either the low-risk or high-risk group based on the median risk score. The distribution of the risk score and survival status among the high-risk and low-risk groups is illustrated in Fig. 3D, E within the training set. The visual depiction offers a comprehensive comprehension of the distribution of risk scores and their correlation with the patients' survival results. Based on the findings from the Kaplan–Meier analysis, it was observed that the high-risk group demonstrated a significantly diminished OS in comparison to the low-risk group, as depicted in Fig. 3F. Furthermore, the ROC analysis provided evidence of the risk score's robust predictive ability in determining the survival status. The ROC curve demonstrated the effectiveness of the risk score in accurately predicting the survival status, as evidenced by the AUC values of 0.715, 0.69, and 0.678 for 1-, 3-, and 5-year survival, respectively (Fig. 3G). The distribution of risk scores and survival statuses within the high- and low-risk groups of the validation set is depicted in Fig. 3H, I. The Kaplan–Meier analysis demonstrated a statistically significant disparity in overall survival between the high-risk and low-risk groups (Fig. 3J). The ROC curve showed that the risk score performed well in predicting survival status with AUC values for 1-, 3-, and 5-year survival of 0.821, 0.754, and 0.731, respectively (Fig. 3K).

### Clinical feature analysis

**Fig. 4:**
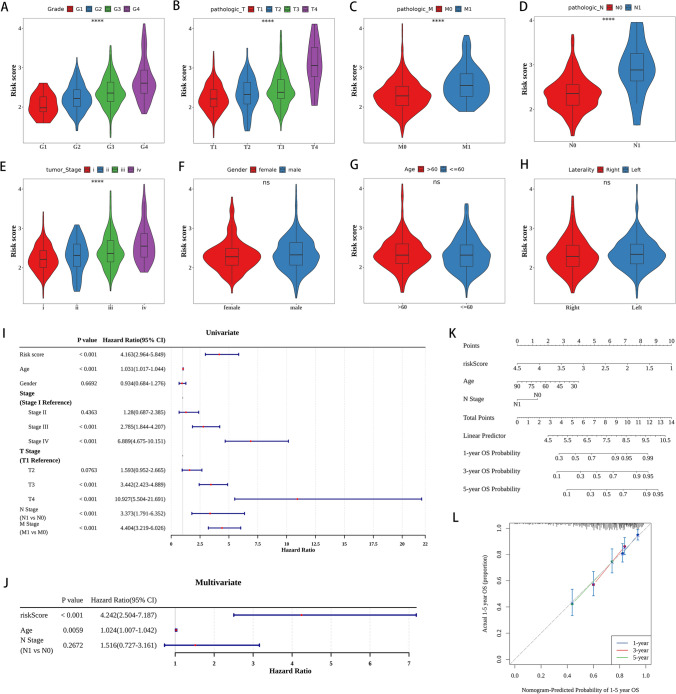
Clinical feature analysis and construction of the nomogram in ccRCC. The relevance between the risk score and clinicopathological traits, including grade (**A**), pathologic T (**B**), pathologic M (**C**), pathologic N (**D**), tumor Stage (**E**), Gender (**F**), age (**G**) and laterality (**H**). **I** Univariate analyses to detect independent prognostic factors. **J** Multivariate analyses to detect independent prognostic factors. **K** Nomogram was established. **L** The calibration curves for 1-, 3-, and 5-year. The data were presented as mean ± SD. ****P < 0.0001

Significant correlations were found when assessing the connections between the risk score and different clinicopathological characteristics, including age, gender, laterality, grade, pathologic T, pathologic M, pathologic N, and tumor stage. Significantly, the risk score showed noteworthy correlations with grade, pathologic T, pathologic M, pathologic N, and tumor stage (Fig. 4A–E). In contrast, there were no notable connections found between the risk score and gender, age, or laterality (Fig. 4F–H).

### Construction of the nomogram in ccRCC

Univariate analysis was conducted using age, sex, risk score, pathologic T, pathologic M, pathologic N, and tumor stage as variables (Fig. 4I). The prognostic model was constructed through the implementation of a multivariate analysis, wherein variables such as risk score, age, pathologic N, and tumor stage were taken into account (Fig. 4J). Based on these factors, a nomogram was developed to estimate the survival probability of ccRCC patients at 1-, 3-, and 5-years (Fig. 4K). Figure 4L displayed strong agreement between the forecasted survival probability from the nomogram and the real survival results for the 1-, 3-, and 5-year durations, as shown by the calibration curves.

### Immune analysis of the high- and low-risk groups

**Fig. 5:**
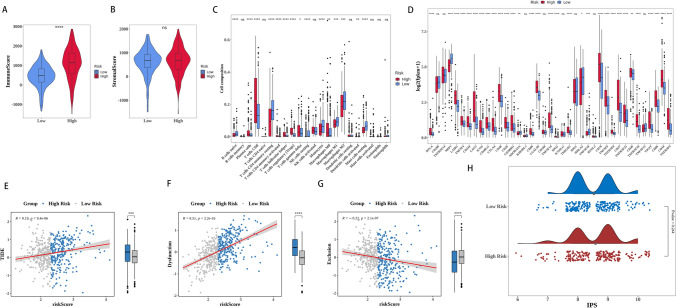
Immune analysis of the high and low risk groups and risk model predictability in immunotherapy response immune analysis of high and low risk groups. Immune (**A**) and stromal (**B**) scores of high and low risk groups. **C** Screening immune cell between the high and low risk groups by CIBERSORT databases. **D** The expression of immune checkpoints between high-risk and low-risk groups. **E**–**G** Association between risk model and immune infiltration. **H** IPS between high and low risk groups. The data were presented as mean ± SD. *P < 0.05, **P < 0.01, ***P < 0.001, ****P < 0.0001

The analysis of the high-risk and low-risk groups showed that the high-risk group had a considerably greater immune score compared to the low-risk group (p < 0.001) (Fig. 5A). Nevertheless, there existed a notable disparity between the two factions (Fig. 5B). By utilizing CIBERSORT, we successfully detected variations in the composition of 15 immune cell categories between the high-risk and low-risk cohorts. The immune cells comprised of inexperienced B cells, plasma cells, T cells with CD8+ markers, T cells with CD4+ markers in a state of memory rest, T cells with CD4+ markers in an activated memory state, T cells that assist in follicular development, regulatory T cells (Tregs), T cells with γδ markers, inactive NK cells, monocytes, macrophages in M0 state, macrophages in M1 state, macrophages in M2 state, dendritic cells in an activated state, and mast cells in a resting state (Fig. 5C).

In addition, various immune checkpoint markers, such as BTLA, NRP1, LAIR1, TNFSF4, CD244, LAG3, ICOS, CD40LG, CTLA4, CD48, CD28, CD200R1, ADOR3DL1, CD80, LGALS9, TNFSF14, IDO2, TMIGD2, HHLA2, TNFSF18, CD70, TNFSF9, TNFRSF8, CD27, TNFRSF25, CD40, TNFRSF18, TIGIT, CD86, CD44, and TNFRSF9, showed significant differences in expression levels, comparing immune checkpoint expression in high-risk and low-risk groups (Fig. 5D).

### Predictive potential of the risk model for immunotherapy response

To enhance our understanding of the predictive capacity of our risk model in anticipating the efficacy of immunotherapy, we conducted an analysis to examine the correlation between the risk model and established indicators of immunotherapy response, such as TIDE and IPS. TIDE scores were strongly correlated with risk scores in the analysis (p < 0.05). Furthermore, we observed a stronger positive association between the risk scores and TIDE dysfunction (R = 0.51, p < 0.05). On the other hand, there was a negative correlation between the risk scores and TIDE exclusion (p < 0.05) (Fig. 5E–G). Furthermore, there was no notable disparity in the IPS observed between the high-risk and low-risk categories. The suggestion is that our risk model may not be directly associated with the efficacy of immune checkpoint inhibitors in this particular situation (Fig. 5H). To summarize, the aforementioned findings indicate that individuals with reduced risk scores are more likely to experience advantages from immunotherapy.

### Potential function of TMSB10 in prognosis prediction, mechanism of immune infiltration, and therapeutic response

**Fig. 6:**
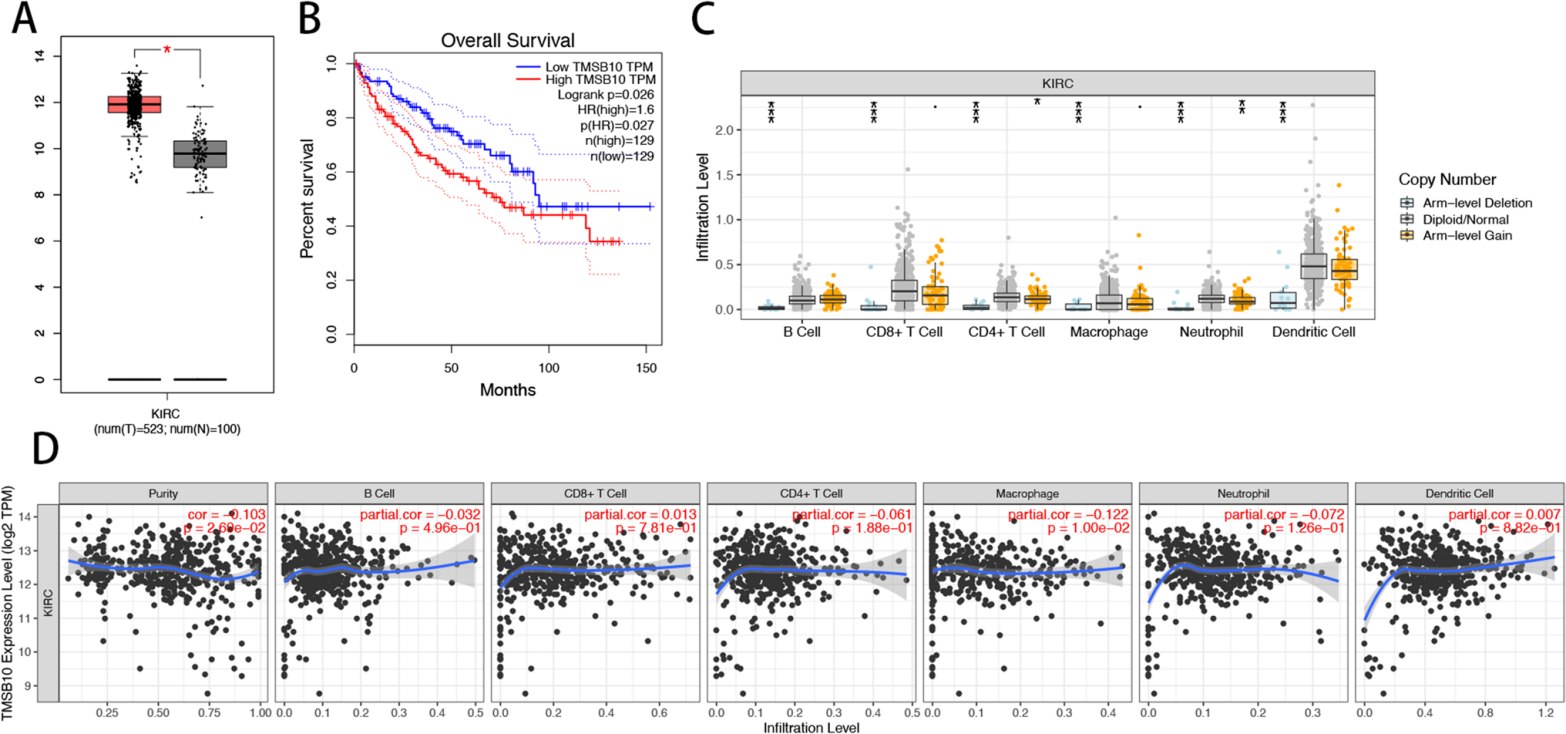
The clinical significance of TMSB10 in ccRCC. The upregulation of TMSB10 in ccRCC samples, as evidenced by data from TCGA (**A**) and cell lines (**B**), is associated with a significantly lower expression level of TMSB10, which in turn is correlated with a favorable prognosis. **C** Additionally, the copy number of immune cells in ccRCC. **D** The correlation analysis of TMSB10 with infiltrating B cells, CD4+ T cells, CD8+ T cells, Macrophages, Neutrophils, and Dendritic cells using TIMER further support these findings. The data were presented as mean ± SD. *P < 0.05, **P < 0.01, ***P < 0.001

Given the unclear prognostic significance of TMSB10 as a gene related to T cells in ccRCC, we conducted further analyses to investigate the biological function of TMSB10.By analyzing TCGA datasets, we discovered a notable increase in the expression of TMSB10 in tumor tissues compared to normal tissues (Fig. 6A). The study on the survival of ccRCC patients with different levels of TMSB10 expression aimed to assess the predictive ability of TMSB10. The results indicated that higher TMSB10 expression (p < 0.05) was linked to shorter overall survival (OS) based on the analysis (Fig. 6B). To elucidate the biological functions of TMSB10 in immune infiltration, we utilized the TIMER database to examine the association between TMSB10 expression and the abundance of infiltrating immune cells. It was discovered that arm-level deletions accounted for the majority of mutations in B cells, CD8+ T cells, CD4+ T cells, macrophages, neutrophils, and dendritic cells (p < 0.001) (Fig. 6C). Moreover, there was a strong correlation between macrophage presence and expression of TMSB10 (p < 0.01) (Fig. 6D). Furthermore, we conducted an assessment of the impact of TMSB10 knockdown on the secretion of immunosuppressive factors TGF-β1 and IL-35 in ccRCC cells using ELISA. The findings indicated that the downregulation of TMSB10 led to a decrease in the secretion of immunosuppressive factors TGF-β1 and IL-35 in ccRCC cells, as illustrated in Figure S1. Consequently, it is suggested that TMSB10 may have a significant involvement in the immune evasion and tumor advancement of ccRCC.

### Effect of TMSB10 downregulation on proliferation, migration, and invasion of ccRCC cells in vitro

**Fig. 7:**
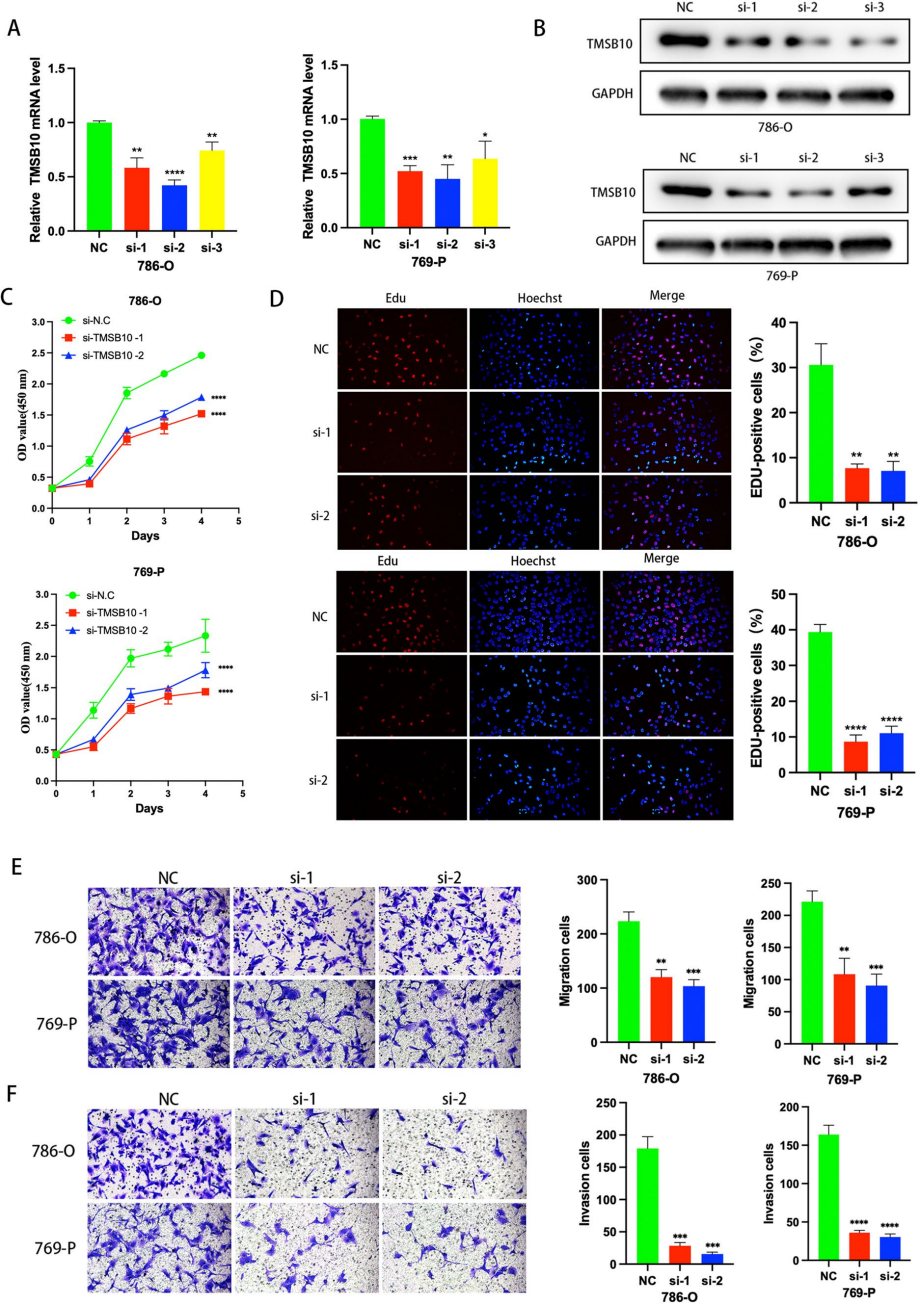
The clinical significance of TMSB10 in ccRCC and in vitro study. **A**, **B** TMSB10 or a negative control siRNA were transfected to verify transfection efficiency. **C** CCK-8 proliferation analysis of the effects of TMSB10 knockdown on the cell growth of 786-O and 769-P at 0, 24, 48, 72 and 96 h post-transfection. **D** EdU assays. **E**, **F** TMSB10 knockdown cells were applied for transwell analysis. Representative images of migrated cells and invaded cells were shown. The indicated migrated and invaded cells were quantified in three randomly chosen fields and presented as mean ± SD. *P < 0.05, **P < 0.01, ***P < 0.001, ****P < 0.0001

We suppressed the expression of TMSB10 mRNA in three ccRCC cell lines to understand the biological role of TMSB10. In qRT-PCR assays and Western blots, siRNA transfection remarkably reduced TMSB10 mRNA and protein expression (Fig. 7A, B). TMSB10 knockdown significantly decreased the proliferation of ccRCC cells in CCK-8 assays (Fig. 7C). The findings from the EdU assay also indicated that the suppression of TMSB10 greatly impeded the growth of ccRCC cells (Fig. 7D). Moreover, the Transwell assay findings unequivocally exhibited a significant reduction in the migratory and invasive capabilities of ccRCC cells after TMSB10 knockdown (Fig. 7E, F). These results demonstrate that the proliferative, migratory, and invasive properties of ccRCC cells are inhibited by TMSB10 knockdown.

## Discussion

In recent years, significant advancements have been made in the field of cancer immunotherapy [23]. Various research investigations have indicated that the existence of immune infiltration contributes to the advancement of ccRCC [24–26], specifically γδ T cells [27], which accountable for various immune reactions in the cancer-causing procedure and function as a vital connection between the innate and adaptive immune systems [28]. Moreover, γδ T cells exhibit notable characteristics such as heightened cytokine secretion and non-restricted antigen recognition beyond the major histocompatibility complex. These features hold significant implications for immunotherapy approaches targeting malignant tumors [29]. Therefore, γδ T cells have the ability to significantly influence both the progression of cancers and the response to therapy. The findings strongly indicate that the stimulation of T-cells greatly influences the development and progression of ccRCC [8, 30]. Thus, γδ T cell-based therapeutic strategies may emerge based on current promising preclinical and clinical study results.

In this study, we discovered 304 differential genes related to γδ T cells. The analysis of their functions revealed their involvement in biological processes and the regulation of T-cell activation. These findings aligned with the outcomes of prior inquiries [8, 30]. Moreover, Rancan et al. The positive effect of activating T-cells in the tumor microenvironment on anti-cancer therapy was found [31]. Exploring essential factors that govern the activation and operation of γδ T-cells in this particular situation may unlock new opportunities for improving anti-cancer therapies. In general, our research adds to an increasing amount of proof that supports the possibility of utilizing the immune system to fight against cancer and emphasizes the need for continuous investigation in this encouraging area.

Subsequently, we utilized univariate and LASSO regression methods to identify 13 indicators linked to the outlook of ccRCC that are specifically associated with γδ T cells. Subsequently, these indicators were utilized to create a risk model and nomogram capable of precisely forecasting ccRCC prognosis. To validate the predictive importance of our risk model, we conducted analyses on ROC curve and Kaplan–Meier. The results showed that individuals identified as high-risk had a significantly shorter lifespan in comparison to those classified as low-risk. Moreover, our risk profile was discovered to be a strong autonomous forecaster of outcome, as demonstrated by both single-variable and multi-variable regression examinations. The findings suggest that our risk model could be useful in predicting the outcome of ccRCC.

Zhang et al. used our risk model, which includes 13 markers associated with prognosis. The study found that the reduction in PLCB2 levels significantly affected the survival of melanoma cells, resulting in enhanced apoptosis via the activation of the Ras/Raf/MAPK pathway [32]. Additionally, studies have suggested that TMSB10 may act as a potential predictive biomarker closely linked to the infiltration and development of blood vessels in lung cancer [33], RCC [34], gastric cancer [35], hepatocellular carcinoma [36], and bladder cancer [37]. According to Zeng et al. Reported that the overexpression of TMSB10 in tumors increased the conversion of M2 and the proliferation of tumor-associated macrophages through the PI3K/Akt signaling pathway, thus promoting tumor growth. The discovery presented a hopeful tumor biomarker for forecasting the prognosis of lung adenocarcinoma and a potential target for treatment [38]. Studies have shown that IL4I1 has the capacity to function as both a marker and a focus for treatment [39, 40]. NUSAP1 is linked to the development of different forms of cancers [41, 42]. Eckert et al. The discovery of NNMT in the stroma has revealed its significant involvement in controlling the progression and development of cancer, making it a potential candidate for therapeutic interventions [43].

Lastly, TMSB10 was included in the final model. In comparison to normal tissues, tumor tissues exhibited significantly elevated expression of TMSB10, a well-known oncogene linked to different forms of cancer, as revealed by our examination of TCGA datasets. The finding underscores the potential role of TMSB10 in tumorigenesis and suggests its viability as a promising target for therapeutic interventions across various cancer subtypes [14–16]. Moreover, our research has revealed a compelling association between heightened expression of TMSB10 and a notable decline in OS rates. The discovery underscores the potential significance of TMSB10 as a prognostic indicator for patient results in different forms of cancer, while also underscoring its therapeutic targeting possibilities. Exploring further into the complex processes by which TMSB10 influences OS may reveal priceless knowledge, leading to the creation of focused treatments designed to improve patient survival rates. The excessive expression of TMSB10 in ccRCC cell lines suggests its capability as a predictive indicator for unfavorable results in ccRCC. To comprehend the biological functions of TMSB10 in immune infiltration, an analysis was conducted to investigate the relationship between the expression of TMSB10 and the abundance of infiltrating immune cells. This investigation made use of the highly informative TIMER database, allowing for a comprehensive comprehension of the complex interaction between TMSB10 expression and immune cell infiltration. To enhance our understanding of how TMSB10 may affect the control of the immune microenvironment in different types of cancer, we initiated an investigation into this correlation. A robust correlation has been identified between TMSB10 and the infiltration of immune cells, while the exploration of TMSB10's role in the domain of cancer biology remains limited. Our findings indicate that silencing of TMSB10 leads to a decrease in the release of immunosuppressive factors in clear cell renal cell carcinoma (ccRCC) cells. Prior research [38] has established that TMSB10 can facilitate the transformation of tumor-associated macrophages (TAMs) into the M2 phenotype and enhance their proliferation via the PI3K/Akt signaling pathway, ultimately fostering tumor progression within the tumor microenvironment. Furthermore, there is compelling evidence [44] suggesting a significant association between aberrant TMSB10 expression in tumor tissues and the infiltration of immune cells in the tumor microenvironment. Our research significantly strengthened the connection of TMSB10 in immune escape and tumor progression in ccRCC. During our ongoing investigation, we noticed that the restraining of TMSB10 led to the curbing of ccRCC cell growth, movement, and infiltration. Hence, it is conceivable that TMSB10 might have a crucial part in facilitating the advancement of ccRCC. Additional comprehensive research is necessary to elucidate the precise role and mechanisms of TMSB10 in the advancement of ccRCC, thereby potentially facilitating the development of more efficacious therapeutic approaches.

In summary, our study successfully identified 13 unique genes that are specifically associated with γδ T cells. Subsequently, these genetic elements were utilized in the creation of a risk prediction model and graphical representation. These predictive tools accurately predicted the prognosis of patients with ccRCC. Our study revealed an association between TMSB10 and immune evasion as well as tumor advancement in clear cell renal cell carcinoma (ccRCC). Furthermore, we identified TMSB10 as a pivotal gene in driving the progression of ccRCC and impacting patient prognosis. In the context of ccRCC, the potential efficacy of inhibiting the expression or activity of TMSB10 as a therapeutic strategy warrants consideration. Therefore, this research offers significant understanding into the advancement of focused treatments.

### Supplementary Information

Below is the link to the electronic supplementary material

Supplementary file1 (TIF 3014 KB)

Supplementary file2 (DOCX 15 KB)

Supplementary file3 (DOCX 14 KB)

## Data Availability

The datasets used and/or analysed during the current study are available from the corresponding author on reasonable request.
