# Supplementary figures and images for: A new prognostic model based on gamma-delta T cells for predicting the risk and aiding in the treatment of clear cell renal cell carcinoma
Source: Discov Oncol. 2024 May 25;15:185. doi: 10.1007/s12672-024-01057-2 (PMC11127908; doi:10.1007/s12672-024-01057-2)

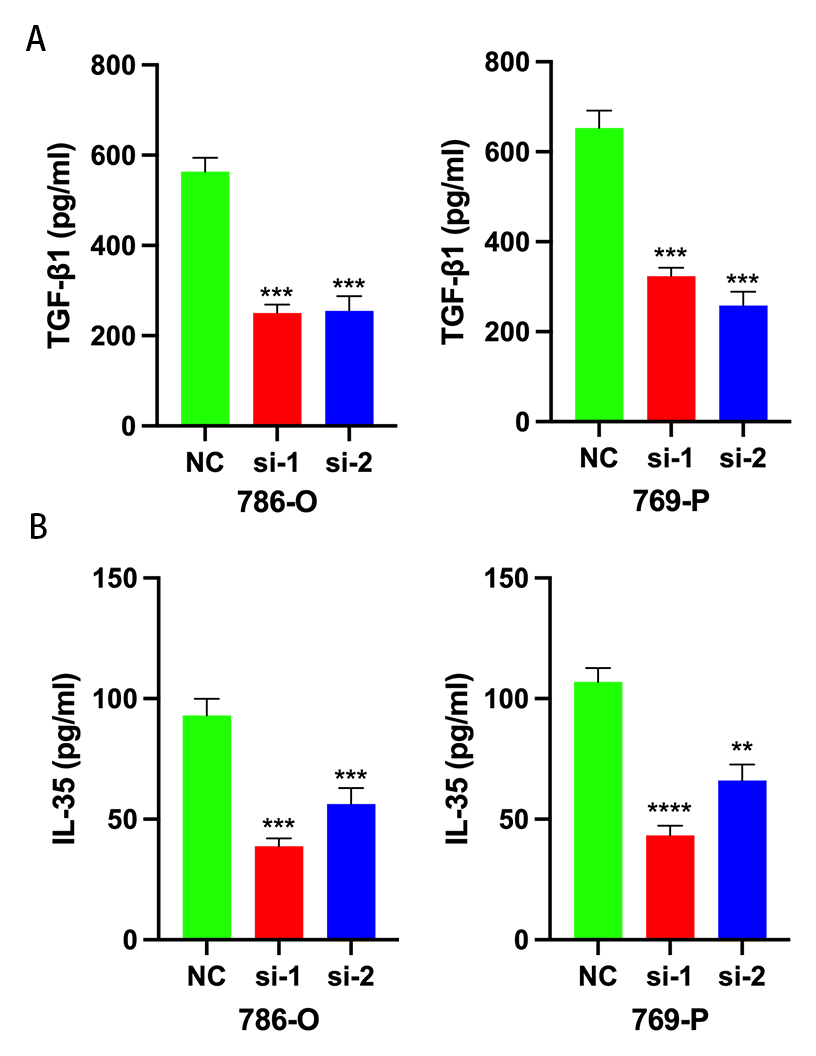

Supplement: Supplementary file 1 — Supplementary file1 (TIF 3014 KB) [file 12672_2024_1057_MOESM1_ESM.tif]
