# Supplementary material for: A new prognostic model based on gamma-delta T cells for predicting the risk and aiding in the treatment of clear cell renal cell carcinoma
Source: Discov Oncol. 2024 May 25;15:185. doi: 10.1007/s12672-024-01057-2 (PMC11127908; doi:10.1007/s12672-024-01057-2)
Supplement: Supplementary file 2 — Supplementary file2 (DOCX 15 KB) [file 12672_2024_1057_MOESM2_ESM.docx]

**Supplementary Figure S1. Knockdown of TMSB10 down-regulated the secretion of immunosuppressive factors TGF****-β1 and IL-35 in ccRCC cells**

(A-B) After TMSB10 knockdown, 786-O and 769-P cells were cultured in complete medium for 48h, and the levels of TGF-β1 and IL-35 secreted by 786 and 769 cells were detected by human TGF-β1 ELISA kit and human IL-35 ELISA kit, respectively. Data are presented as the mean ±SD. **P < 0.01, ***P < 0.001, ****P < 0.0001.
