# Supplementary material for: A new prognostic model based on gamma-delta T cells for predicting the risk and aiding in the treatment of clear cell renal cell carcinoma
Source: Discov Oncol. 2024 May 25;15:185. doi: 10.1007/s12672-024-01057-2 (PMC11127908; doi:10.1007/s12672-024-01057-2)
Supplement: Supplementary file 3 — Supplementary file3 (DOCX 14 KB) [file 12672_2024_1057_MOESM3_ESM.docx]

Supplementary Materials and Methods

**ELISA**

After TMSB10 knockdown in ccRCC cells, the cells were cultured with complete medium for 48h. Finally, the supernatant was collected and analyzed. TGF-β1(Transforming Growth Factor Beta 1) ELISA Kit (E-EL-0162, Elabscience, Wuhan, China) and Human IL-35(Interleukin 35) ELISA Kit (E-EL-H2443, Elabscience, Wuhan, China) were used to determine the concentrations of TGF-β1 and IL-35, respectively, according to the manufacturer’s instructions.
